# Identification of ipsilateral supraclavicular lymph node metastasis in breast cancer based on LASSO regression with a high penalty factor

**DOI:** 10.3389/fonc.2024.1349315

**Published:** 2024-02-02

**Authors:** Haohan Zhang, Jin Yin, Chen Zhou, Jiajun Qiu, Junren Wang, Qing Lv, Ting Luo

**Affiliations:** ^1^ West China Hospital, Sichuan University, Chengdu, China; ^2^ West China Biomedical Big Data Center, West China Hospital, Sichuan University, Chengdu, China; ^3^ Med-X Center for Informatics, Sichuan University, Chengdu, China; ^4^ Division of Breast Surgery, Department of General Surgery, West China Hospital, Sichuan University, Chengdu, China; ^5^ Breast Center, West China Hospital, Sichuan University, Chengdu, China; ^6^ Clinical Research Center for Breast Diseases, West China Hospital, Sichuan University, Chengdu, China; ^7^ Department of Medical Oncology, Cancer Center, West China Hospital, Sichuan University, Chengdu, China

**Keywords:** breast cancer, ipsilateral supraclavicular lymph node metastasis, radiomics, genetic algorithm, LASSO regression, feature combination

## Abstract

Aiming at the problems of small sample size and large feature dimension in the identification of ipsilateral supraclavicular lymph node metastasis status in breast cancer using ultrasound radiomics, an optimized feature combination search algorithm is proposed to construct linear classification models with high interpretability. The genetic algorithm (GA) is used to search for feature combinations within the feature subspace using least absolute shrinkage and selection operator (LASSO) regression. The search is optimized by applying a high penalty to the L1 norm of LASSO to retain excellent features in the crossover operation of the GA. The experimental results show that the linear model constructed using this method outperforms those using the conventional LASSO regression and standard GA. Therefore, this method can be used to build linear models with higher classification performance and more robustness.

## 1 Introduction

The incidence and mortality of female breast cancer in China have been continuously increasing in recent years, posing a large threat to women’s health (1, 2). The survival outcomes of patients with ipsilateral supraclavicular lymph node (ISLN) metastasis after surgery are often unsatisfactory. Identifying ISLN status before surgery allows for categorizing patients. ISLN patients may benefit from initial treatments such as neoadjuvant chemotherapy (3) rather than surgery. Furthermore, breast ultrasound is a routine diagnostic method for breast cancer. This approach is noninvasive, inexpensive, real-time, portable, and radiation-free and provides internal structure imaging that contains rich information on tumor heterogeneity. However, this information is usually difficult to observed visually (4, 5). Radiomics refers to the conversion of medical images into mineable data and the subsequent analysis of these data to provide decision support (4, 5). Therefore, preoperative ultrasound based radiomics technology is an effective noninvasive approach to identify ISLN status before treatment and assist doctors in making clinical decisions.

The relatively low occurrence of ISLN events (4%) results in a limited number of samples that can be collected, especially for single centers (6). Predicting ISLN using an ultrasound radiomics-based approach is evidently a classical data mining problem with a small sample size. Therefore, methods such as deep convolutional neural networks that require a large amount of data and annotations may not be suitable for this study (7, 8). Radiomic feature engineering combined with linear classification models may achieve exceptional performance in terms of model robustness and interpretability (7, 9, 10). To date, there have been no studies on ISLN identification based on preoperative ultrasound radiomics. However, relevant studies have been reported. In 2019, Liu et al. (11) used dynamic contrast enhanced magnetic resonance imaging (DCE-MRI)-based radiomic feature-trained linear logistic regression (LR), extreme gradient boosting (XGBoost), and support vector machine models to identify axillary lymph node metastasis in breast cancer patients. In 2020, Qiu et al. (12) built an LR model based on ultrasound radiomic features to identify axillary lymph node metastasis in breast cancer patients. In the same year, Yu et al. (13) constructed an LR model based on the radiomic features of MRI to identify axillary lymph node metastasis and predicted the disease-free survival of patients with early breast cancer. In the above studies, the clinical problems to be solved were all data mining problems with a small sample size and high-dimensional feature space, and after feature selection, machine learning models were constructed for classification. Further, these studies employed the least absolute shrinkage and selection operator (LASSO) method for feature selection. Then, linear models were constructed to evaluate the classification performance. Although nonlinear models may offer better the training performance, they often have limited generalizability and poor interpretability, making it challenging to apply them in clinical practice. A feasible way to alleviate this issue is to use linear combinations with interpretability to build and train models (9, 10). Currently, the vast majority of radiomics studies use t tests or U tests in combination with correlation coefficients for preliminary feature selection to obtain stable candidate features. Then, conventional (widely used) LASSO regression methods are used for further selection of the candidate features (9, 14).

However, the feature space of datasets in radiomics research is generally a hyperdimensional space composed of thousands of radiomic features, and finding a suitable combination of a finite number of features remains a challenging task (9, 10). LASSO (15) is a linear regression algorithm that can intuitively and explicitly express the representation ability of a feature set. LASSO adds a regularization term to the general linear regression to ensure the best fitting error and to keep the parameters as simple as possible (reducing the number of nonzero parameters) to reduce overfitting. Therefore, LASSO can enable a machine learning model to exhibit strong generalizability and outstanding robustness (16, 17). However, as a multivariate regression method that searches for a feature subset from the feature space, LASSO first searches the entire feature space. When the dimension of the feature space is very high, the choice of the optimal penalty factor lambda (i.e., the weight of the L1 norm or penalty value/penalty factor) can be challenging. A high lambda can keep the parameters as simple as possible, thereby improving the robustness of the constructed linear model. However, the model performance may decrease. On the other hand, a low lambda value may lead to overfitting the model constructed in the next step. Liu et al. (9) proposed preselecting features based on statistical testing, i.e., preselecting candidate features. In this process, first, the total dimensionality of the feature space is reduced by defining a feature subspace in advance. Then, LASSO is used to search for the optimized feature combination within this subspace. This approach is widely used in radiomics studies (9, 10). Nevertheless, on the one hand, the predefined feature subspace may still be a high-dimensional feature space. On the other hand, LASSO only searches for feature combinations within this feature subspace, which is still redundant to include more candidate features. A more optimized solution is to further extract subspaces in this feature subspace multiple times and use LASSO to search for feature combinations in each of these extracted subspaces. To ensure completeness, extracted subspaces must cross each other, a requirement similar that when sampling with replacement.

This study aims to address the issue of small sample size and high-dimensional feature space in the identification of the ISLN metastasis status of breast cancer patients by ultrasound radiomics. To this end, we combine the genetic algorithm (GA) with LASSO and propose GALambda, in which the crossover operation of individuals is carried out based on LASSO regression with a high penalty factor. In this approach, the GA is used to extract feature subspaces as well as evaluate and iterate over these extracted subspaces to determine if LASSO can find feature combinations that can construct high-performance linear prediction models within these subspaces. Furthermore, for the GA, a strategy is designed to dynamically set excellent genes based on the high penalty parameter lambda. Thus, the excellent genes are preserved for crossover to generate offspring in the hope that the search for feature combination can be optimized.

## 2 Materials and methods

### 2.1 Dataset

#### 2.1.1 Study population

Female patients diagnosed with primary breast cancer with ISLN metastasis at West China Hospital between December 2010 and May 2020 were retrospectively included in this study. The diagnosis of breast cancer and ISLN metastasis was confirmed by preoperative biopsy or postoperative pathological examination of ISLN specimens. The inclusion criteria were as follows: (1) female patients with primary breast cancer; and (2) patients who underwent preoperative ISLN biopsy or postoperative pathological examination of ISLN specimens. The exclusion criteria were as follows: (1) lack of ultrasound image records; and (2) negative ISLN biopsy results without postoperative pathological records. A total of 181 patients were finally included in this study. These patients were randomly divided into a training set (n=109) and an independent test set (n=72) at a ratio of 6:4. The results of the statistical tests indicated that there were no significant differences in important baseline features (age, T-stage, invasive ductal carcinoma, progesterone receptor status, estrogen receptor status, and intraductal carcinoma *in situ* of the breast) between the training and test sets (p≥ 0.05). In the statistical test of significant differences, Fisher’s exact test was used for categorical variables and the Mann–Whitney U test (also known as the Mann–Whitney rank sum test) was used for continuous variables. The p values for the above six baseline features were 0.7256, 0.3979, 0.1322, 0.4391, 0.2079, and 0.4429, respectively.

#### 2.1.2 Ultrasound images

All patients in this study underwent preoperative breast and lymph node ultrasound. The ultrasound equipment used were as follows: an Acuson S3000 with an 18-5 MHz linear transducer (Siemens, Munich, Germany) and an iU22 with a 12-5 MHz probe (Philips, Amsterdam, Netherlands). The stored ultrasound images should (1) be from the most recent ultrasound examination before ISLN status confirmation through biopsy or dissection, (2) contain the entire tumor or lymph node lesions and as many features as possible, (3) clearly show the tumor lesions, and (4) be obtained by an experienced sonographer. Two sonographers with more than 10 years of experience in breast ultrasound outlined primary lesions as the region of interest (ROI) on the ISLN ultrasound images.

### 2.2 Methods

**Figure 1:**
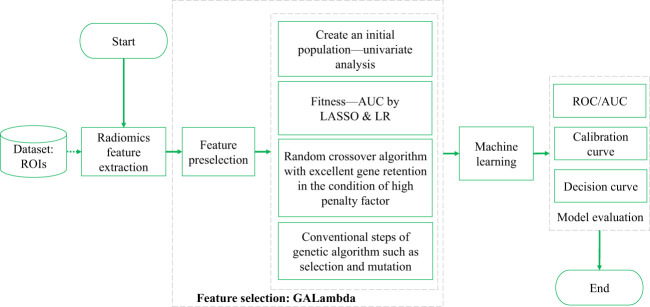
Technical route of identifying ISLN by ultrasound radiomics.

The technical route of this study, which is also the conventional technical roadmap of radiomics, is shown in **Figure 1**. Both feature selection and training were performed on the training set. A five-fold cross-validation (CV) strategy was used during the training. The model evaluation was primarily based on the results from CV and independent testing. GALambda is an improved GA proposed in this study based on LASSO regression that obtains the features corresponding to nonzero coefficients by selecting a high penalty factor from the LASSO CV results. GALambda dynamically sets excellent genes for the crossover operation on individuals in the GA based on the minimum mean square error from CV. As a result, the features that are retained under the high penalty factor in each combination from multivariate LASSO regression can be carried over to the new individuals generated. This algorithm will be described in more detail in the subsequent section.

#### 2.2.1 Radiomic feature extraction

Quantitative radiomic features were extracted from the ROIs and the subbands of filtered ROIs. The filtering methods included the Laplacian of Gaussian (LoG), wavelet transform, and contourlet transform methods. The quantitative feature extraction methods included the histogram, cooccurrence matrix, run-length matrix, difference matrix, size-zone matrix, neighborhood difference matrix, and neighborhood dependency matrix methods. Shape features were also extracted. The above methods were applied again to the subbands of the filtered ROIs, and there were a total of 25 feature extraction methods, resulting in a total of 5031 features extracted from a single ROI (18–20).

#### 2.2.2 GALambda: GA and LASSO regression

**Figure 2:**
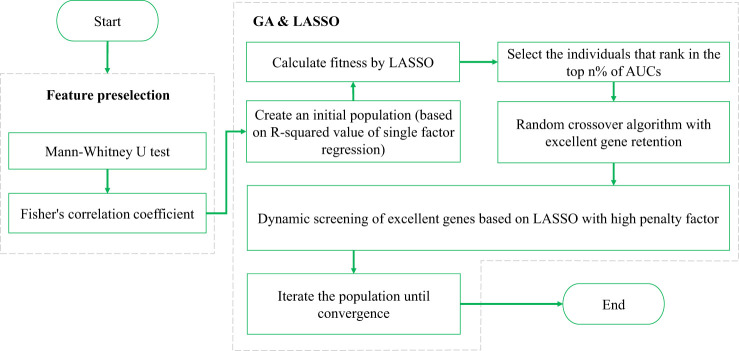
Basic framework of the GALambda method.

In feature selection, the GA can be used to search randomly and in parallel for a feature combination in the feature space so that the objective cost (i.e., fitness) of the feature combination is minimized. However, standard GAs randomly converge and fail to effectively control the generalizability of the feature combination. On the other hand, the LASSO regression is characterized by variable screening and complexity adjustment (regularization, L1 norm) while fitting a generalized linear model. The L1 norm of LASSO regression constrains, adjusts, or shrinks the coefficient estimates towards zero, so LASSO regression can be used for feature selection. Besides, when the penalty factor for the L1 norm is larger, the feature corresponding to the nonzero coefficient can play a more important role in multivariate regression. As the lambda value increases, the number of nonzero coefficients gradually decreases. However, LASSO regression searches for which features are more important (controlled by the size of the lambda value) for the regression result within a specific combination. Therefore, the regression performance depends not only on the choice of lambda but also obviously on the combination to be searched. In general, conventional LASSO regression selects lambda based on the minimum mean square error of k-fold CV. However, if LASSO regression is used to screen for variables from a large feature space, this approach can still result in too many nonzero coefficients, causing overfitting. If it is possible to select variables from an appropriate feature space, the number of nonzero coefficients can be reduced. However, a search in a specific feature space may limit the results obtained (which potentially leads to a local optimal result). Therefore, the plan in this study is to screen for variables in two steps based on the principles of the GA: the first step is the feature subspace search, and the second step is LASSO regression within the identified feature subspace. The specific framework is shown in **Figure 2**.

First, feature preselection is performed based on the Fisher correlation coefficient and statistical U test to obtain candidate features (9, 10). In the U test, a p-value less than 0.05 is commonly considered a significant difference statistically. The Fisher correlation coefficient approach is to calculate the pairwise correlation coefficients of features, and a threshold of 0.8 is empirically chosen. When the correlation coefficient is greater than or equal to 0.8, the two features are strongly correlated, and the feature with a higher average correlation is removed (9).

Then, LASSO and its lambda configuration are combined in the GA as follows:

1. The gene expression of an individual is represented as a binary string, with a length equal to the number of candidate features. Here, ‘0’ indicates that the corresponding feature is not selected, while ‘1’ indicates that the corresponding feature is selected. In this context, ‘selected’ refers to being included in a feature subspace, rather than being in the final feature result. LASSO regression is then applied to this feature subspace to obtain the final feature results.
2. After performing LASSO regression with k-fold CV on the feature subspace, the features with a lambda value less than the minimum mean standard error and nonzero coefficients are the finally selected features. The fitness function is located as the 1-area under the curve (AUC) value of the k-fold CV after performing a linear logistic regression on the final selected features.
3. Crossover operation. The crossover operation is applied to the genes of two individuals to generate new individuals. The main idea of the GALambda method for crossover is as follows. When searching for feature combinations in the feature subspace specified by an individual, the features corresponding to nonzero coefficients that are still retained under the penalty parameter lambda can be retained, while the remaining genes are randomly selected from their parents. This process enables the excellent genes that are retained even under high penalty parameters in LASSO regression to be directly inherited by the next generation as features in the feature subspace specified for the next generation, allowing feature combinations in the next generation to still include these features in the search. This also ensures diversity in the feature subspace, maximally mitigating the issue of the feature search becoming too localized in a single feature subspace. The specific pseudocode for the crossover operation is shown in **Algorithm 1**.

Algorithm 1 **Pseudocode for the crossover operation in GALambda.**

**
*Input:*
nvars:** number of genes, i.e., the number in the feature
space;
**Bs:** pop-by-1 cellular data, where pop is the population
size. Bs{i} represents the coefficients corresponding
to each lambda returned by executing LASSO on an
individual i, and it is a nvars-by-nlambdas matrix,
with the j-th column being the coefficient
corresponding to j-th lambda;
**Fs:** pop-by-1 cellular data. Fs{i} represents the
fitting information returned by executing LASSO on
individual i, including the mean square error (MSE)
vector and the lambda index/location corresponding to
the minimum mean square error (indexMinMSE);
**a, b:** both are 1-by-nvars vectors, the features are
arranged from 1 to nvars, with an element value of 0 or
1, where 1 means that the corresponding feature is
selected and 0 means that the corresponding feature is
not selected
**ia, ib:** denote the locations of individuals a and b in the
population, respectively
**
*Output:*
offspring:** 1-by-nvars vector.
1 **Begin:**
2 //Step 1/5: List of the predefined number of
  excellent genes and the average mean square error
3 nums = [ … ];//list of the number of excellent genes
4 mses = [ … ];//list of the average mean square error
  corresponding to nums
5 na = 1; nb = 1;//Numbers of excellent genes for
  individuals a and b, with default values of 1
6 //Step 2/5: Dynamically set the number of excellent
  genes according to the LASSO average mean square
  error
7 **for** i=1 **to** length (mses) {
8 if Fs{ia}.MSE(Fs{ia}.indexMinMSE)<= mses(i) {9 na/nb = nums(i); break};
10 }
11 //Step 3/5: Screen for excellent genes based on a
  high penalty lambda (refer to **Algorithm 2**).
12 excellentGeneAIndices=screenExcellentGenes(Bs
  {ia}, a);
13 excellentGeneBIndices=screenExcellentGenes(Bs
  {ib}, b);
14 //Step 4/5: Set the index of excellent genes to 1,
  indicating that a new individual has been selected
15 excellentGeneIndices = unique
  ([excellentGeneAIndices,
  excellentGeneBIndices]);
16 offspring = zeros(1, nvars);//A new individual
17 offspring(excellentGeneIndices)=1;//set
  excellentgenes
18 //Step 5/5: Randomly select other genes from their
  parents (half from each parent)
19 offspring = combine(new Individual, a, b);
20 **End**

In **Algorithm 1**, lines 6-13 are the mechanism for dynamically setting excellent genes; lines 14-17 set excellent genes for new individuals; and lines 18-19 randomly assign other genes from parental individuals to new individuals.

**Algorithm 2** is the pseudocode that first screen for excellent genes of the parental individuals in the crossover process, that is, the mechanism that dynamically sets the excellent genes according to the lambda parameter and fitness in the LASSO regression of the individual to be crossed over. The search starts from the maximum lambda value and stops when the number of nonzero coefficients meets the value specified by the input parameter. The input parameter n is determined by the predefined fitness interval in the crossover (refer to lines 2-5 of **Algorithm 1**).

Algorithm 2 **Pseudocode for screening for excellent genes based on high penalty parameters.**

**
*Input***:
**B:** nvars-by-nlambdas matrix, where nvars is the number
of genes (the number of features, i.e., the dimension of
the feature space), nlambdas represents the number of
penalty parameter lambda, and lambda is sorted in
ascending order from the first to the last column in B;
B is obtained by executing LASSO, so the value of the
excellent genes screened must be 1, indicating that the
corresponding feature is selected;
**n:** The features are arranged from 1 to nvars, with the
elements in a or b having values of 0 or 1, where 1 means
that the corresponding feature is selected and 0 means
that the corresponding feature is not selected.
**
*Output***:
**geneIndices**: 1-by-n vector, expressing the locations of
excellent genes.
1 **Begin**:
2 //Lambda starts to search from the maximum value and
  stops when the number of nonzero coefficients is
  greater than or equal to n
3 **for** j=width(B):-1:1
4 //v is a vector coefficient of the j-th lambda
5 v = abs(B(:j));
6 v = sort(v, ‘descend’);//Sort v in descending order
7 indices = find(v~=0);//Features corresponding to
  nonzero coefficients
8 **if** length (indices) >= n {
9 geneIndices = indices (1:n);
10 return gene Indices};
11 }//end for: End of loop for traversing lambda
12 **End**

For a given lambda value, the minimization problem to be solved by LASSO regression is defined in Equation 1, where N is the number of training samples, *i* and *j* are the sample numbers, and x and y represent the sample (feature vector) and response (i.e., the outcome variable, which in this study is either 1 for the presence of metastasis or 0 for the absence of metastasis), respectively, λ is the regularization parameter, that is, the penalty parameter lambda, which is nonnegative, β is the coefficient vector corresponding to the feature, and b is the intercept scalar. As can be observed from the last term of Equation 1, the lambda parameter is actually the weight of the L1 norm/regularization as a penalty factor/parameter. In regression, the contribution of a feature to the total score is noted with a nonzero coefficient value, but a larger penalty factor reduces the values of less important coefficients, possibly even down to 0, thus removing the feature corresponding to the coefficient from the regression.


(1) $$\text{min}\left(\frac{\sum_{i=1}^{N}{\left(y_{i}-b-x_{i}^{T}\beta \right)}^{2}}{2N}+\lambda \sum_{j=1}^{M}\left|\beta_{j}\right|\right)$$

In addition, considering that the GALambda method searches for linear combinations of features from the feature subspace, if the dimension of the feature subspace in the initial population is too low, GALambda may search in the sparse feature subspace. However, the method itself has the capability to sparsify the feature subspace through lambda tuning. Therefore, the GALambda method generates an initial population based on the univariate regression results. Specifically, the value of the regression evaluation metric R-squared in univariate regression is converted to a probabilistic expression—the gene being 1 (greater than or equal to 0.5) or 0 (less than 0.5). R-squared, as defined in Equation 2, is also known as coefficient of determination and is an important metric for assessing how well the coefficients fit the true value of y. In Equation 2, y represents the true value, the symbol ˆ denotes the predicted value, the symbol ˉ denotes the average value, and N is the number of samples to be fitted/trained.


(2) $$R^{2}=1-\frac{\sum_{i=1}^{N}{\left(\hat{y}-y_{i}\right)}^{2}}{\sum_{i=1}^{N}{\left(y_{i}-\bar{y}\right)}^{2}}$$

## 3 Experimental results and discussion

The features selected by GALambda are shown in **Table 1**. A one-level wavelet decomposition can produce an approximate component that represents low-frequency information and three detail components that represent horizontal, vertical, and diagonal high-frequency information, respectively. The approximate component can be decomposed again. A one-level contourlet transform consists of a pyramid decomposition and a directional filter decomposition, which can produce an approximate component that represents low-frequency information and 2^n^ (decided by the directional filter bank) detail components. The approximate component can be decomposed again. Specifically, (1) the wavelet transform is carried out with three-level decomposition; the method names have the prefix CMS-W; feature extraction is performed on nine high-frequency components, where components 1, 2, and 3 are obtained from the first-level decomposition, components 4, 5, and 6 are obtained from the second-level decomposition, and components 7, 8, and 9 are obtained from the third-level decomposition; and (2) the contourlet transform is performed with the three-level decomposition; the method names have the prefix CMS-C; eight high-frequency components are obtained from the first-level decomposition, and four components are each obtained from the second-level and third-level decomposition. The components of contourlet decompositions are similarly numbered as the wavelet decompositions.

**Table 1:** Features selected by GALambda.

Method name	Feature name	Parameter
FOD	10th percentile	
GLRLM	Low gray-level run emphasis	
CMS-WRLM	Long run emphasis	component = 5
CMS-WSZM	Zone entropy	component = 4
CMS-WSZM	Low gray-level zone emphasis	component = 4
CMS-WSZM	Size-zone nonuniformity normalized	component = 6
CMS-WNDM	Dependency variance	component=1 | distance=3
CMS-WNDM	Dependence entropy	component=9 | distance=3
CMS-WNTM	Strength	component=6 | distance=1
CMS-WNTM	Complexity	component=6 | distance=3
CMS-CFOD	Kurtosis	component=16
CMS-CCOM	Correlation	component=14 | distance=3
CMS-CSZM	Size-zone nonuniformity normalized	component = 2
CMS-CSZM	Gray-level variance	component=10
CMS-CSZM	Size-zone nonuniformity normalized	component=14
CMS-CNTM	Strength	component=9 | distance=1

**Figure 3:**
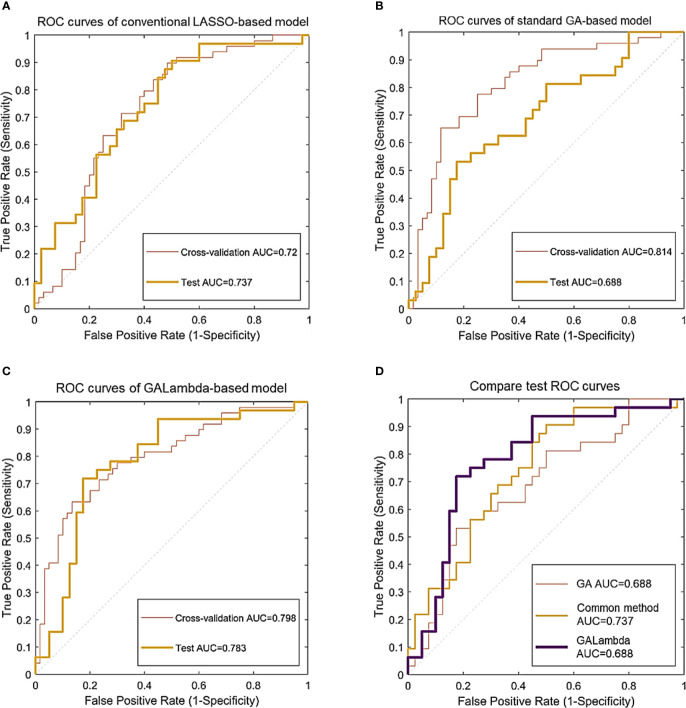
Classification ROC curves for the linear models constructed using feature combinations obtained by three methods. **(A)** ROC curves of conventional LASSO-based model. **(B)** ROC curves of standard GA-based model. **(C)** ROC curves of GALambda-based model. **(D)** Compare test ROC curves.

GALambda first performed feature preselection based on thresholds of Fisher’s correlation coefficient and the Mann–Whitney U test. Then, GALambda performed the genetic algorithm as described in section 1.2.2 on the preselected features. As for an individual, LASSO regression was performed on the feature space corresponding to this individual. As described in **Algorithms 1** and **2**, high penalty factors were set to screen excellent genes and then a customized crossover operation was performed. After population iteration, 16 features were finally selected. Logistic linear regression was performed based on the selected features, and predictions were made on the independent test set. The AUC and the accuracy corresponding to the maximum Youden index were calculated. The results are shown in **Table 2** (the values in parentheses represent the 95% confidence interval based on the sampling strategy with replacement—100 calculations), and the corresponding ROC curves are shown in **Figure 3**. Considering that previous relevant studies (11–13) have used LASSO to select features, and most radiomics studies also usually applied LASSO for feature selection (9, 10), a conventional LASSO method was also implemented in the experiments of this study. Besides, GALambda searches for linear combinations of features in random subspaces delineated by a genetic algorithm. For comparison, the standard genetic algorithm is also implemented to select features. The selected features by conventional LASSO and standard GA are shown in the **Supplementary Materials**.

**Table 2:** AUCs and accuracies for CV and the test results.

	CV AUC	CV accuracy	Test AUC	Test accuracy
Conventional method	0.720 (0.631-0.828)	0.688 (0.607-0.773)	0.737 (0.644-0.851)	0.681 (0.584-0.789)
Standard GA	0.814 (0.729 - 0.903)	0.780 (0.688 - 0.853)	0.688 (0.560 - 0.820)	0.694 (0.609 - 0.788)
**This work**	**0.798 (0.702 - 0.896)**	**0.761 (0.684 - 0.849)**	**0.783 (0.668 - 0.890)**	**0.778 (0.701 - 0.876)**

Bold values means the classification results of the GALambda method model.

In the results of **Table 2** and **Figure 3**, all three methods are based on the search for linear combinations of features based on the preselected candidate features. In the medical literature, 1/10 or 1/5 of the training sample size is widely used as the lower limit of feature number for developing prediction models that predict a binary outcome, which also generally been accepted as a methodological quality item in appraising published prediction modeling studies (14). To further reduce overfitting, the maximum number of nonzero coefficients was limited in LASSO regression. This value was empirically set to 1/10 or 1/5 of the training sample size. The conventional method optimizes the parameters for feature preselection during the experiment, that is, a grid search was conducted for the thresholds of the statistical significance tests and Fisher’s correlation coefficient. A p-value less than 0.05 in the U test is commonly considered a significant difference statistically, and Fisher’s correlation coefficient is greater than or equal to 0.8 is commonly considered that the corresopnidng two features are strongly correlated (9, 10). Therefore, we developed a small threshold grid centered at 0.05 and 0.8 for the U test and Fisher’s correlation coefficient respectively, and used the cross-validation AUC as the evaluation criterion to select the optimal thresholds. **Table 2** shows that the Galambda-based model achieves higher test AUC and accuracy than the models of the other two methods. Correspondingly, as shown in **Figure 3**, the test ROC curve of the GALambda-based method approaches the upper left corner of the figure faster than the other two curves.

Although the standard GA did identify a linear combination of features with the highest CV AUC, its robustness is poor because the standard GA lacks an effective regularization parameter to control overfitting. The linear combination of features identified by the conventional method exhibited lower CV and test AUCs than the GALambda method. The same comparison results were also performed in terms of accuracy. This may be attributed to the fact that it only conducts a global search within a large feature subspace. In contrast, GALambda, because of its combination with the GA, initially searches for feature subspaces and can obtain a good feature subspace before performing LASSO regression, resulting in a more focused and richer search for linear combinations of feature and thereby ultimately leading to the discovery of a linear combination of features that can better predict lymph node metastasis. In addition, confidence intervals account for the variabilities in results. As for CV AUCs of standard GA and GALambda, the upper bounds of 95% confidence intervals are almost close to each other (0.903 vs. 0.896). As for CV accuracies of standard GA and GALambda, the lower bounds of 95% confidence intervals are almost close to each other (0.688 vs. 0.684). It may indicate that the model of GLambda would be no worse than the model of standard GA in the training.

**Figure 4:**
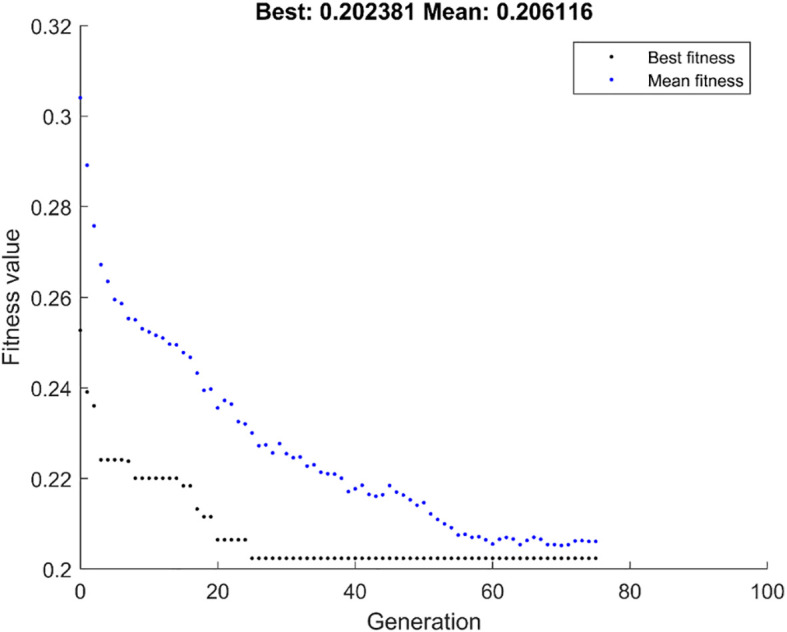
The population iterative process using GALambda.

**Figure 5:**
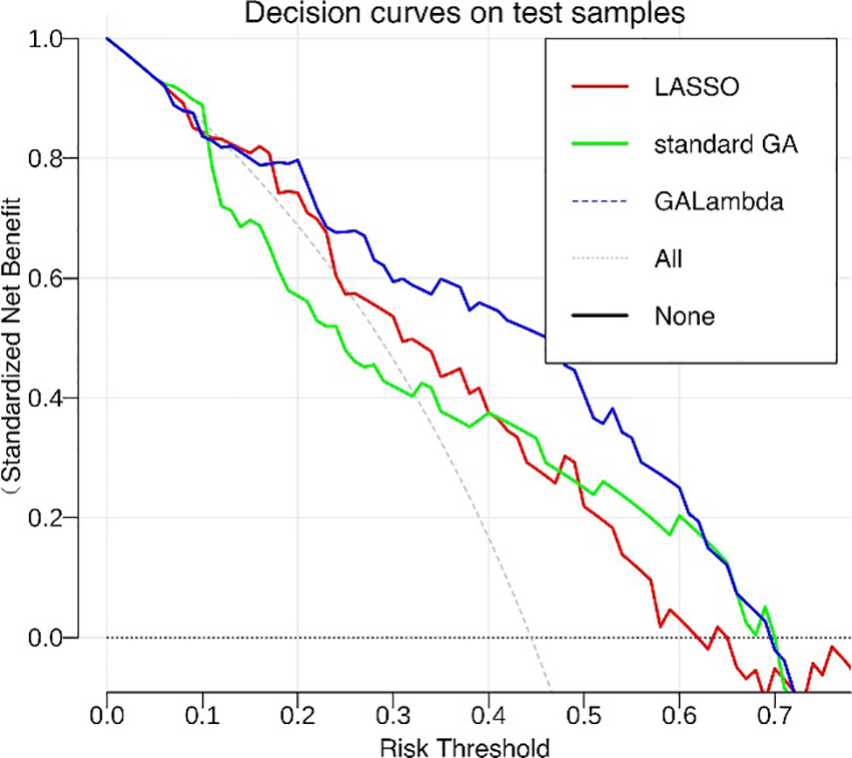
Decision curves of linear models constructed by feature combinations obtained using three methods.

**Figure 4** shows the population iteration process of the GALambda algorithm, which gradually converges after approximately 75 iterations. The convergence is relatively fast and the complexity of the GA is not significantly increased. To further evaluate the clinical utility of the linear combination of features optimized by the GALambda algorithm in the construction of a linear model to predict lymph node metastasis, decision curves were used to assess the utility of the constructed linear model for guiding clinical intervention. For predictive models related to clinical problems, decision curves are also a crucial metric for assessing the model robustness. The decision curves in **Figure 5** show that the linear model constructed based on the feature combination obtained by GALambda for the prediction of lymph node metastasis to guide the interventions can achieve a higher net benefit than those of the other two methods over a broader range of risk thresholds, suggesting that the linear model constructed by the feature combination obtained using the GALambda method has greater robustness and clinical utility. Besides, prediction models such as linear combinations such as regression are generally considered effective means of reducing overfitting. However, this has high criteria for the number of training samples and variables. Generally, the smaller the number of variables relative to the number of training samples, the lower the potential overfitting of a model (14). GALambda screens features in random subspaces divided by a genetic algorithm, thus screening out fewer features, which helps reduce overfitting. The experimental results also showed that the GALambda-based model performs the best on the independent test.

However, this study also has some limitations. First, it is a single-center study, and an external validation set usually preferably reflects the robustness and generalization ability of models, which can further verify the superiority of GALambda. Then, GALambda is a method based on random search. The final result is related to an initial state. Initialization population can affect the searching in subspaces and the subsequent evolution. Future studies can try various initialization methods to optimize GALambda.

## 4 Conclusion

In this study, to address the problems of small sample size and high-dimensional feature space of datasets to predict ISLN metastasis for breast cancer patients using ultrasound radiomics, we explored a GA, referred to as GALambda. GALambda performs a crossover operation dynamically set by LASSO regression based on a high penalty factor to optimize the search for feature combinations and construct linear prediction models with high interpretability. The experimental results showed that the GALambda method exploits the advantages of the GA by first searching for a feature subspace and then performing regularized LASSO regression to identify feature combinations within this feature subspace. In addition, based on the minimum mean square error through CV in LASSO regression, the GALambda method dynamically selects the features corresponding to nonzero coefficients under a high penalty factor for individual crossover in the GA, obtaining better feature combinations than those obtained using the conventional LASSO method and the standard GA after a finite number of population iterations. The evaluation of the AUCs of the ROC and decision curves showed that the linear prediction model constructed based on the feature combination obtained by the GALambda method could obtain better prediction results, which is helpful for the construction of prediction models of ISLN metastasis for breast cancer patients using ultrasound radiomics. In addition, this method also has value in quantitative data mining in other radiomics studies.

## Data availability statement

The data analyzed in this study is subject to the following licenses/restrictions: Currently, researchers can obtain a fully anonymized dataset by emailing the corresponding authors if the researchers declare that the data are only used for research purposes.. Requests to access these datasets should be directed to Haohan Zhang, zhanghaohan@stu.scu.edu.cn.

## Author contributions

HZ: Writing – original draft. JY: Writing – original draft. CZ: Writing – review & editing. JQ: Writing – review & editing. JW: Writing – review & editing. QL: Writing – review & editing. TL: Writing – review & editing.
