# Supplementary material for: Identification of ipsilateral supraclavicular lymph node metastasis in breast cancer based on LASSO regression with a high penalty factor
Source: Front Oncol. 2024 Feb 2;14:1349315. doi: 10.3389/fonc.2024.1349315 (PMC10869533; doi:10.3389/fonc.2024.1349315)
Supplement: Supplementary file 1 [file DataSheet_1.pdf]

Identification of ipsilateral supraclavicular lymph node metastasis in breast cancer based on LASSO regression with a high penalty factor

Supplementary Materials

Metric for Fitness Function

The AUC is a major evaluation metric for features fitting into a model, and its calculations are shown in Equations (1) to (3).

$$TPR = \frac{TP}{TP + FN} \tag{1}$$

$$FPR = 1 - \frac{TN}{TN + FP} = \frac{FP}{TN + FP} \tag{2}$$

$$AUC = \int_0^1 tpr(x) \times dx \tag{3}$$

TPR is the true positive rate, TP is the number of true positives, FN is the number of false-negatives, FPR is the false-positive rate, FP is the number of false-positives, and TN is the number of true negatives. In Equation (3), x represents the FPR value calculated according to Equation (2) when a threshold point is set and tpr(x) represents the TPR value at that threshold point (i.e., the TPR value when the FPR is x). By setting several threshold points in the range of 0 to 1, tpr(x) forms the receiver operating characteristic (ROC) curve.

Selected Features by Conventional LASSO

Method name	Feature name and parameter
FOD	10th percentile
CMS-WCOM	correlation|component=8|distance=2
CMS-WSZM	low gray level zone emphasis|component=4
CMS-WSZM	size-zone non-uniformity normalized|component=6
CMS-WNDM	dependence entropy|component=9|distance=3
CMS-WNTM	complexity|component=6|distance=3
CMS-CSZM	size-zone non-uniformity normalized|component=2
CMS-CSZM	gray level variance|component=10
CMS-CSZM	size-zone non-uniformity normalized|component=14

A one-level wavelet decomposition can produce an approximate component that represents low-frequency information and three detail components that represent horizontal, vertical, and diagonal high-frequency information, respectively. The approximate component can be decomposed again. A one-level contourlet transform consists of a pyramid decomposition and a directional filter

decomposition, which can produce an approximate component that represents low-frequency information and 2^n (decided by the directional filter bank) detail components. The approximate component can be decomposed again. Specifically, (1) the wavelet transform is carried out with three-level decomposition; the method names have the prefix CMS-W; feature extraction is performed on nine high-frequency components, where components 1, 2, and 3 are obtained from the first-level decomposition, components 4, 5, and 6 are obtained from the second-level decomposition, and components 7, 8, and 9 are obtained from the third-level decomposition; and (2) the contourlet transform is performed with the three-level decomposition; the method names have the prefix CMS-C; eight high-frequency components are obtained from the first-level decomposition, and four components are each obtained from the second-level and third-level decomposition. The components of contourlet decompositions are similarly numbered as the wavelet decompositions.

Selected Features by Standard GA

Method name	Feature name and parameter
FOD	10th percentile
CMS-WCOM	correlation|component=9|distance=3
CMS-WSZM	zone entropy|component=1
CMS-WSZM	low gray level zone emphasis|component=4
CMS-WSZM	size-zone non-uniformity normalized|component=6
CMS-WNDM	dependence variance|component=1|distance=3
CMS-WNDM	dependence entropy|component=9|distance=3
CMS-WNTM	strength|component=6|distance=1
CMS-WNTM	complexity|component=6|distance=3
CMS-CFOD	kurtosis|component=16
CMS-CCOM	correlation|component=14|distance=3
CMS-CNTM	strength|component=9|distance=1
CMS-CNTM	strength|component=14|distance=1
